# A Collaborative Framework Toward a Transparent and Structured Pre-submission Peer Review Process in Scientific Publishing

**DOI:** 10.5152/tud.2026.26059

**Published:** 2026-10-23

**Authors:** Ayad Palani, Asli Metin Mahmutoglu, Taymour Mostafa, Keerti Singh, Hiva Alipour, Germar-Michael Pinggera, Walter D. Cardona Maya, Mohamed Arafa, Erman Ceyhan, Tan V. Le, Damayanthi Durairajanayagam, Mohammed Abdulgabbar Noman, Ramadan Saleh, Rupin Shah, Ates Kadioglu, Rossella Cannarella, Ashok Agarwal

**Affiliations:** 1 Global Andrology Forum, Global Andrology Foundation, Moreland Hills, OH, USA; 2 Department of Basic Sciences, College of Medicine, University of Garmian, Kalar, Iraq; 3 Department of Clinical Biochemistry, Zanko Central Laboratory, Kalar, Iraq; 4 Department of Medical Biology, Yozgat Bozok University Faculty of Medicine, Yozgat, Türkiye; 5 Department of Andrology, Sexology and STIs, Cairo University, Cairo, Egypt; 6 Department of Preclinical and Health Sciences, The University of the West Indies Faculty of Medicine, Cave Hill Campus, Bridgetown, Barbados; 7 Department of Health Science and Technology, Aalborg University, Aalborg, Denmark; 8 Department of Urology, Medical University of Innsbruck, Innsbruck, Austria; 9 Reproduction Group, Department of Microbiology and Parasitology, University of Antioquia Faculty of Medicine, Medellín, Colombia; 10 Department of Urology, Hamad Medical Corporation, Doha, Qatar; 11 Department of Urology, Weill Cornell Medicine-Qatar, Doha, Qatar; 12 Department of Urology, Başkent University, Ankara, Türkiye; 13 Science Research and Training Department, Binh Dan Hospital, Ho Chi Minh City, Vietnam; 14 Department of General Surgery, Pham Ngoc Thach University of Medicine, Ho Chi Minh City, Vietnam; 15 Department of Physiology, Universiti Teknologi MARA Faculty of Medicine, Selangor, Malaysia; 16 Department of Dermatology and Venereology, Sana’a University, Sana’a, Yemen; 17 Department of Dermatology, Venereology and Andrology, Sohag University Faculty of Medicine, Sohag, Egypt; 18 Ajyal IVF Center, Ajyal Hospital, Sohag, Egypt; 19 Department of Urology, Lilavati Hospital and Research Centre, Mumbai, India; 20 Department of Urology, İstanbul School of Medicine, İstanbul University, İstanbul, Türkiye; 21 Department of Clinical and Experimental Medicine, University of Catania, Catania, Italy; 22 Cleveland Clinic, Cleveland, OH, USA

**Keywords:** Editorial policies, GAF model, open access publishing, peer review, publishing

## Introduction

Scientific publishing plays a central role in advancing science by disseminating knowledge and sharing new findings among researchers.^1^ In recent decades, the development of the World Wide Web has profoundly transformed the scientific publishing system and reshaped the scholarly communication landscape.^2^ This transformation has led to an exponential increase in published articles. A search of articles and reviews indexed in the Scopus database using the search terms “DOCTYPE (ar) OR DOCTYPE (re)” shows an increase from 3488 publications in 1868 to more than 3.5 million articles in 2025. This rapid expansion has been associated with structural shifts in research dissemination, particularly the proliferation of new publishing channels, including online journals, open access (OA) models, conference proceedings, and journal “special issues,” collectively contributing to increased publication output.^3,4^ Importantly, OA models play a crucial role in making innovative research more accessible, diverse, and widely disseminated.^5^

However, certain publishers exploit the OA model by establishing journals primarily to collect inflated article processing charges (APCs).^6^ Examples of these journals include mega-journals, which publish extremely large numbers of articles annually,^7,8^ and predatory journals, which often promise rapid processing of manuscripts.^9^ By placing greater emphasis on quantity than quality, such journals usually accept a large number of submitted manuscripts, often with minimal or no peer review, to generate APC revenue.^6^ These global challenges in scientific publishing are particularly pronounced in specialized fields, such as andrology, where high-quality evidence is limited and clinical guidance remains debated.

Furthermore, the growing pressure to publish for academic promotion has led some researchers to prioritize publication quantity over manuscript quality. Meanwhile, scholarly journals continue to strengthen their standards through increased selectivity. This situation has led to higher rejection rates, which may reach 85%-90% in some high-impact journals.^10^ This situation highlights the importance of rigorous pre-submission review to ensure high manuscript quality before journal submission.

With the rise in the number of manuscripts submitted through online publishing systems, the editorial process has become increasingly challenging due to a growing reluctance of expert reviewers to participate in editorial evaluations, particularly among leading experts.^11^ Such reluctance can be attributed to several factors, including time constraints (especially for volunteer reviewers with busy schedules), the lack of financial compensation, insufficient recognition for review work, and scientifically weak manuscripts.^12,13^ As a result of these difficulties in recruiting qualified reviewers, editors often invite less experienced reviewers, which is concerning because reviewers with insufficient expertise are not equipped to evaluate manuscripts adequately. Such situations may lead either to the publication of scientific articles containing fundamental methodological flaws that less experienced reviewers fail to identify, or to the rejection of sound scientific ideas because reviewers become overly critical in an attempt to be thorough.^11^ Additionally, reliance on author-nominated reviewers may bias the peer review process.^14^

These challenges have spurred interest in collaborative, structured pre-submission review models designed to enhance transparency, accountability, and scientific rigor. This editorial discusses the major limitations of contemporary peer review and presents a pre-submission review framework as a proposed model to improve fairness, consistency, reviewer accountability, and scientific rigor in scholarly publishing.

## 1. Challenges in Contemporary Peer Review

### 1.1. Transparency and Accountability in Peer Review

Peer review remains the cornerstone of scholarly publishing, serving as the primary mechanism for ensuring the validity, originality, and scientific rigor of academic research.^15^ However, despite its foundational role, the traditional peer review system has come under increasing scrutiny for limited transparency, opaque editorial decision-making, and inconsistent accountability.^11,16,17^ In this regard, transparency in peer review means making the evaluation process visible by openly sharing reviewer comments, author responses, and editorial decisions so that the basis on which research is judged becomes clear and accountable. In response to these concerns, there have been sustained calls to explore alternative review models to enhance fairness and structural equity.^18,19^

Journal systems currently use several peer review models, depending on the level of anonymity for authors and reviewers. These include peer reviews that are either single-blinded (authors’ identities are known to the reviewers, while the reviewers are anonymized) or double-blinded (both authors and reviewers are anonymized).^20-22^ While these models aim to reduce identity-based bias, implementation remains challenging because of practical constraints, including the difficulty of fully masking author identity in highly specialized fields and the administrative complexity of maintaining strict anonymity.

To address these limitations, the open peer review model has emerged as a strategy to enhance transparency and accountability in manuscript evaluation. In some journals, reviewer reports and authors’ responses are published alongside the article, providing greater insight into editorial decision-making.^23^

Collaborative peer review models have been introduced in recent years to facilitate structured dialog among reviewers, and in some cases between reviewers and authors, with the aim of encouraging constructive critique and shared intellectual responsibility.^24^ While these innovations promote openness and accountability, they may also influence reviewer frankness. Publicly visible reports can discourage critical feedback, and increased interaction may introduce strategic behavior or reinforce power imbalances. Thus, transparency alone does not automatically guarantee fairness or objectivity; governance structures must be carefully designed to balance openness with independence.

### 1.2. Bias, Fairness, and Reviewer Diversity

Bias in scholarly publishing is multifactorial and embedded within broader academic hierarchies. Various forms of bias may undermine the objectivity, fairness, and efficiency of peer review. These can be broadly classified into 2 categories: conscious (explicit) bias and unconscious (implicit) bias, with the latter being more prevalent and often operating unnoticed.^24^

In general, 2 major categories of bias are commonly described in the peer review system:

1. **Cognitive Bias**: Reviewers’ preconceptions heavily influence outcomes, meaning the exact same paper could be accepted by 1 journal and rejected by another.^25^
2. **Prestige and Institutional Bias**: There is a tendency to favor work from prestigious institutions or well-known authors, geographic location, and language background, sometimes penalizing novel or disruptive research from lesser-known scholars, thereby reinforcing systemic imbalances.^24^

Additionally, gender-related inequalities have also been documented in the peer review process. Data from the Nature Portfolio Diversity Commitment illustrate this imbalance: women accounted for only 25% of reviewers of research articles in *Nature Microbiology*. Although *Nature Metabolism* reports an increase in female peer reviewers from 18.1% in 2020 to 32.4% in 2024, a substantial gender gap still persists.^25^ These trends reflect incremental progress but underscore enduring disparities. A systematic review identified measurable gender bias in scientific publishing and concluded that while double-blind review may mitigate some effects, it does not eliminate structural inequities.^26^ Addressing these issues requires not only procedural refinement but also sustained institutional commitment to inclusivity and accountability.

### 1.3. Quality, Rigor, and Timeliness of Review

The quality, rigor, and timeliness of peer review form the triad of scientific publishing. These factors ensure research validity and prompt dissemination. They are critical to academic publishing and evidence-based decision-making. In this context, operational pressures further threaten the sustainability of peer review. Reviewer fatigue, along with increasing difficulties in recruiting and incentivizing qualified experts, has contributed to growing delays in manuscript processing.^16^ Such delays are particularly problematic in medical research, where the timely dissemination of findings is essential for improving treatments and ultimately benefiting patients, caregivers, and other researchers in the field. Extended review timelines may also slow the circulation of scholarly knowledge and impose significant constraints on authors’ academic progress.^27,28^ Evidence from a systematic review indicates substantial variation in publication timelines across biomedical journals. The mean time from manuscript submission to acceptance ranges from 11 to 362 days, and from submission to publication from 91 to 639 days. Editorial handling times also vary widely, ranging from a few months to nearly 2 years, thereby delaying the dissemination of new scientific findings.^27^

Simultaneously, rapidly increasing submission rates continue to intensify pressure on journals to balance methodological rigor with timely publication. Under these sustained workload constraints, the consistency and thoroughness of peer review may decline. Addressing these challenges requires sustainable reforms that reconcile efficiency with critical evaluation, ensuring that efforts to accelerate publication do not compromise scientific integrity.

### 1.4. Ethical Standards in Peer Review

Ethical concerns further complicate the peer review landscape. Undisclosed conflicts of interest (whether financial, professional, or personal) can undermine confidence in editorial decisions and threaten perceptions of fairness and integrity.^29,30^ To safeguard credibility, journals must adopt transparent disclosure requirements, standardized reporting practices, and clear recusal procedures to strengthen accountability within the review process.^31^

### 1.5. Artificial Intelligence Technologies in Peer Review

Emerging technologies, particularly generative artificial intelligence (AI), are increasingly being integrated into editorial workflows to improve efficiency and consistency.^15^ AI tools may assist with preliminary manuscript screening, plagiarism detection, reporting checks, and reviewer matching. However, peer review remains a critical intellectual process that requires scientific expertise, ethical judgment, and contextual interpretation.^32^

During the preparation of this manuscript, Gemini was used to assist with language editing and graphic illustration. All scientific content, interpretations, and conclusions were independently reviewed and finalized by the authors.

Although AI may support administrative aspects of peer review, its integration also raises important ethical and methodological concerns.^33^ Potential risks include breaches of manuscript confidentiality, AI-generated critiques lacking contextual or disciplinary judgment, algorithmic hallucinations, propagation of inaccurate citations, and loss of human accountability in scholarly evaluation.^11,34-36^ Therefore, technological innovation alone cannot resolve the complex structural challenges facing peer review, and sustainable reform requires governance frameworks that integrate transparency, accountability, and responsible human oversight.

Potential risks associated with integrating AI in peer review include the following:^11,34-36^

- Reviewers upload confidential manuscripts into AI systems without the authors’ consent.
- AI-generated critiques may lack contextual, disciplinary, or clinical judgment.
- Loss of human accountability and intellectual ownership.
- Algorithmic hallucinations or incorrect methodological interpretations.
- Generating and hence propagating wrong citations.

Table 1 summarizes key challenges in the review process and the corresponding Global Andrology Forum (GAF) recommendations.

**Table 1.** Key Challenges in Scientific Publishing and Peer Review Processes, with the Corresponding Recommendations from the GAF

Publishing and Systematic Issues	Key Challenge	Underlying Causes	Potential Risk	Impact on Scientific Quality	Recommended Solutions by GAF
OA journals	- High APCs.	- Commercialization of OA publishing.	- Reduced insistence on major revisions, particularly when authors are willing to pay APCs. - Editorial fatigue, where financial sustainability becomes intertwined with manuscript throughput.	- Potential acceptance of low-quality manuscripts “pay-to-publish.” - Limited publication opportunities for high-quality research from low-income regions due to high APCs.	- Transparent APC policies. - Consideration of waivers or escrow models for the accepted articles. - Separation of financial and editorial processes. - Public disclosure of editorial independence policies.
Mega-journals	- Rapid increase in the volume of scientific publications. - Reviewer fatigue, due to frequent declines by experienced experts, may force editors to rely on less experienced reviewers.	- Rapid publication sought by some authors.	- Lowered rejection thresholds for manuscripts with moderate methodological flaws. - Expansion of “predatory” or “quasi-predatory” journals, where acceptance rates are intentionally high.	- Publishing low-quality articles.	- Transparent publishing policies. - Transparent reporting of acceptance rates.
Predatory OA journals	- Imitating reputable journal names. - Providing fake indexing information.	- Deceiving authors into paying for fake or illegitimate publishing.	- Absence of a proper editorial and peer review system.	- Authors do not receive thorough or constructive editorial reviews, which compromises the quality and rigor of the published work.	- Avoid journals with unclear origins and verify indexing in reputable databases, such as Scopus or Web of Science.^37^
Reviewer fatigue	- Overreliance on senior experts. - Limited participation of early-career researchers. - Time constraints and competing academic responsibilities. - Increased demand for qualified reviewers with emergence of new journals.	- Concentration of review requests among senior experts. - Rising manuscript submission volumes. - Limited reviewer time due to academic workloads.	- Reviewer fatigue and increased review delays. - Editorial backlog and longer peer review timelines. - Superficial reviews and higher invitation rejection rates. - Use of artificial intelligence for review.	- Bias and potential rejection of high-quality research. - Loss of diverse perspectives and potential rejection of quality studies. - Review delays slowing the circulation of scholarly knowledge. - Superficial reviews constraining authors’ academic progress.	- Formal reviewer training to improve review quality.^38^ - Clear and transparent reviewer selection processes. - Expanded reviewer databases with diverse, qualified experts.
Unethical use of AI in peer review	- AI tools cannot replace domain-specific critical evaluation. - The peer review system risks become mechanistic and unreliable. - AI’s extensive use of manuscripts for critique can lead to a feedback loop of both automated production and evaluation that may be detached from genuine scholarly scrutiny.	- Widespread availability. - Fulfilling the task in a short time.	- Reviewers upload confidential manuscripts into AI systems without the author’s consent. - AI-generated critiques are lacking contextual clinical judgment. - Superficial reviews produced through prompt-based summarization.	- Loss of human accountability and intellectual ownership. - Algorithmic hallucinations/incorrect methodological interpretations. - Risk of data privacy breaches.	- Clear prohibition of uploading manuscripts to external AI platforms (https://www.icmje.org/). - Mandatory disclosure of AI use in peer review (https://www.icmje.org/). - Defined boundaries between AI-assisted editing and AI-generated evaluation. - Human accountability as a non-negotiable principle (https://www.thelancet.com/peer-review). - Ethical governance frameworks for AI integration.
Manuscript rejection	- Wasting of the editors’ and reviewers’ time during the peer review process.	- Lack of originality, methodological errors, ethical concerns or poor writing quality.	- Author fatigue due to manuscript rejection, particularly following recurrent rejections of the same manuscript.	- Poor writing quality could delay publication of studies with important findings.	- Comprehensive and rigorous pre-submission peer review.

AI: artificial intelligence; APCs: article processing charges; GAF: Global Andrology Forum; OA: open access.

While several reforms and alternative peer review approaches have been proposed, no single model has been universally validated as a comprehensive solution to the structural limitations of peer review. Accordingly, the framework presented below should be considered a proposed, evolving collaborative model rather than a definitive replacement for conventional editorial peer review systems.

### 1.6. Manuscript Rejection: Editorial and Peer Review Perspectives

High-quality scientific work is key to successful publication in peer-reviewed journals.^39^ However, reviewers or editors may reject a manuscript when they determine it is low quality, limiting its scientific value. Several deficiencies may contribute to manuscript rejection, including lack of novelty, methodological flaws, poorly argued literature reviews, plagiarism, and poor writing.^40,41^ A well-designed, well-written manuscript is essential to increase the likelihood of acceptance and reduce the risk of rejection.

## 2. A Structured Collaborative Pre-submission Review Framework

### 2.1. Establishing a Pre-submission Review Framework

The GAF is an international online andrology research collaborative group with diverse representation of clinicians and researchers from around the world.^42^ The GAF members assume different roles to create a systematic framework for research projects aimed at producing high-quality articles. The GAF has established a standardized, auditable online review model to strengthen fairness, transparency, and scientific rigor (Supplementary File 1). This structured, multi-reviewer, consensus-driven framework constitutes an internal pre-submission review process designed to optimize the quality of the submitted manuscript. Supplementary File 2 presents a sample of reviewers’ responses from the internal review.

The framework preserves author anonymity throughout the review process while incorporating structured and consistently applied evaluation criteria to minimize subjectivity, discretionary variation, and implicit (unconscious) bias. A defining component of the model is the structured assessment of reviewers’ contributions and formal recognition based on the level of intellectual input. By integrating a structured review framework with a quantitative scoring system, the model promotes rigorous, consistent, and constructive evaluations. The GAF’s internationally diverse membership across 97 countries and multiple related disciplines further reinforces its robustness by enhancing contextual awareness, reducing regional bias, and encouraging collaboration between senior and early-career experts in a transparent, accountable review environment.

Participating in peer review is a rewarding and valuable experience that all researchers are encouraged to engage in. It is a learned skill set that requires knowledge of study design, critical appraisal, ethical considerations, and general expertise in a field of study.^43^

### 2.2. Guiding Principles of the Review Framework

To ensure fairness and rigor in the internal pre-submission review process, the following procedure and guiding principles are applied:

- All manuscripts are treated as privileged documents and protected as intellectual property.
- All identifying details of the author(s) are removed to minimize bias during the review process.
- Inviting several subject matter experts as reviewers to evaluate a manuscript to improve the quality of the article.
- All reviewers complete a standardized online evaluation form assessing the manuscript's clarity, methodological rigor, and scientific merit within the scheduled deadline.
- The use of AI tools in peer review is prohibited, preserving human accountability.

### 2.3. Authorship and Review Oversight Model (Figure 1)

1. **Lead Author’s Role**: Reviews are compiled, considered in full, and addressed in a point-by-point rebuttal. When reviewers provide substantially conflicting or incompatible recommendations, the senior author(s) evaluate the divergent opinions and provide guidance to facilitate a balanced, consistent, and scientifically justified resolution. At this stage, the lead author(s) know the reviewers’ identities. Authors may accept or reject comments, but must provide written justifications. After finalizing the revision, the lead author responds individually to each reviewer, explaining how every comment was addressed or providing a clear justification when a recommendation was not accepted. This ensures transparency and reviewer satisfaction throughout the collaborative review process.
2. **Reviewer Evaluation**: Each review is independently evaluated by the lead author and 2 other designated team members and graded on a scale of 1-5 based on its depth, clarity, methodological insight, and scientific value. The scoring system is intended to promote accountability and recognize meaningful scholarly contributions. In selected cases, reviewers whose contributions involve substantial intellectual critique, interpretation, and manuscript development may be considered for co-authorship in accordance with the International Committee of Medical Journal Editors (ICMJE) authorship criteria, while other meaningful contributions may be acknowledged appropriately.
3. **Integrity Oversight**: GAF management monitors authorship recognition and intervenes if unfair practices are suspected.

### 2.4. Limitations and Unresolved Challenges

Despite its potential advantages, this collaborative pre-submission review framework has several limitations. The process may increase the revision burden for authors because manuscripts frequently receive extensive comments from multiple reviewers. Coordinating reviewers and maintaining timely responses may also become administratively demanding, particularly in large multidisciplinary collaborations. Furthermore, the framework has not yet been externally validated across multiple specialties or publishing environments. Additional concerns include potential reviewer-hierarchy effects, an overemphasis on internal consensus, and uncertainty about scalability beyond structured collaborative research groups. Accordingly, the framework should be viewed as an evolving model requiring further refinement and independent evaluation.

**Figure 1.**
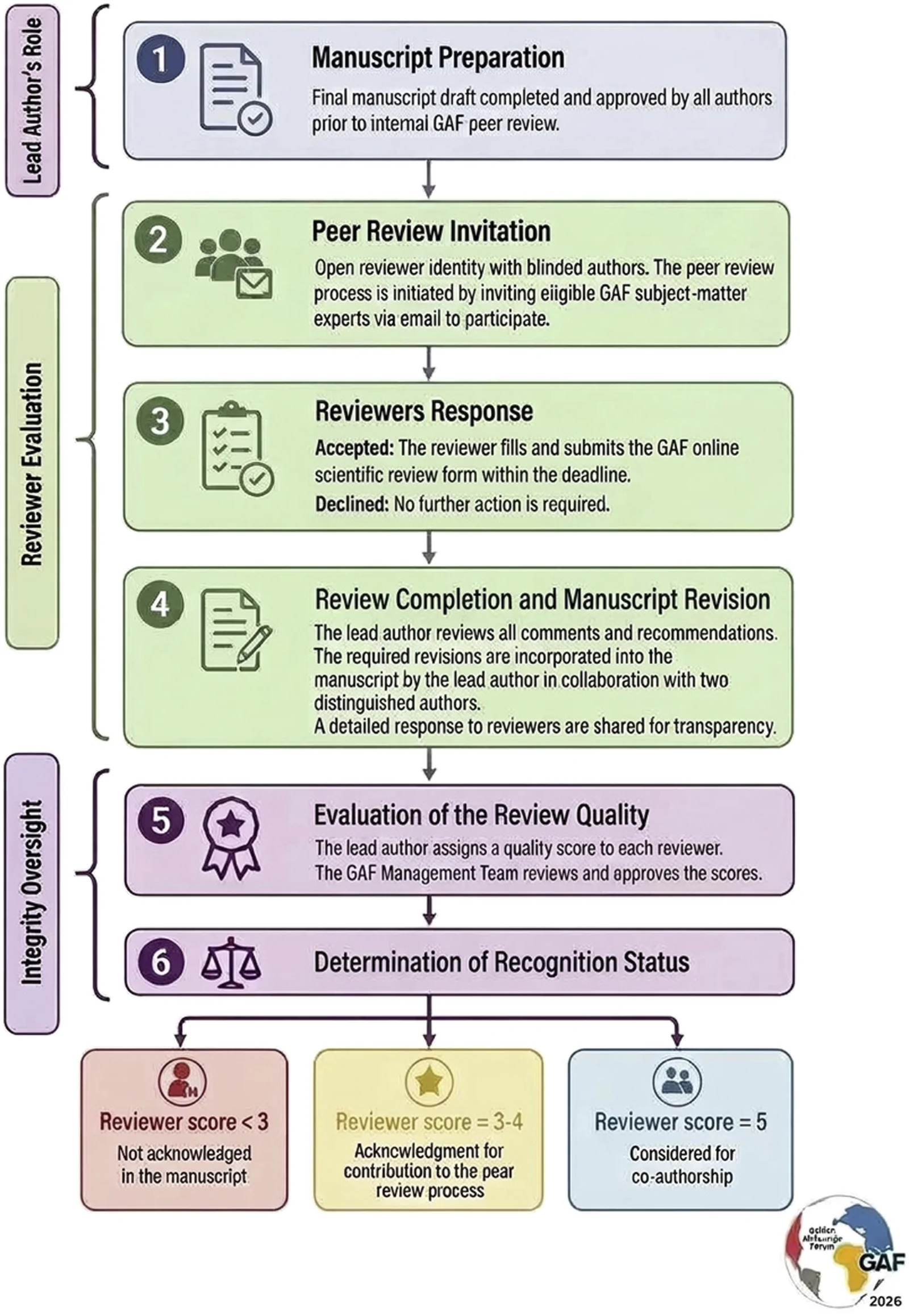
Authorship and reviewer oversight model.

## 3. Potential Advantages of Structured Collaborative Pre-submission Review

The GAF pre-submission peer review model ensures a rigorous evaluation process in which manuscripts are critically reviewed by both the primary authors and independent global experts. This collaborative, multi-author approach strengthens scientific rigor, broadens perspectives, and ensures that the published work is balanced, transparent, and trustworthy. By incorporating structured review forms, strict anonymity, and reviewer grading, the GAF model

- improves manuscript quality before journal submission;
- broadens article impact by integrating diverse global expertise;
- enhances the reviewer recognition system; and
- provides a potentially reproducible framework for transparency, accountability, reviewer recognition, and scientific rigor.

## Conclusion

Peer review remains the cornerstone of scientific publishing, yet contemporary systems continue to face important challenges related to transparency, reviewer variability, accountability, and increasing workload pressures. The structured, collaborative pre-submission review framework presented in this editorial is a proposed GAF model intended to strengthen methodological rigor, reviewer accountability, and transparency in collaborative research environments.

## Data Availability

The data that support the findings of this study are available on request from the corresponding author.
